# Acceleration of performance recovery and competitiveness through non-banking financing in SMEs based on green economy: impact of Covid-19 pandemic

**DOI:** 10.1186/s13731-021-00166-4

**Published:** 2021-07-14

**Authors:** Sriyono, Sarwendah Biduri, Bayu Proyogi

**Affiliations:** Universitas Muhammadiyah Sidoarjo, Sidoarjo, Indonesia

**Keywords:** Acceleration recovery, Performance, Competitive running, Non-banking financing, Competitive excellence

## Abstract

Many previous studies have examined the effects of Covid-19 on small-medium enterprises but never discussed how to accelerate small-medium enterprise performance. This study aims to accelerate the recovery of small-medium enterprise performance affected by Covid-19. This research type is interpretive qualitative, data validity test using credibility and transferability test, data analysis technique using research data reduction, presenting data, and drawing conclusions. The conclusions obtained in this study are financing coming from non-banks when the Covid-19 pandemic conditions are very beneficial for small-medium enterprises, and small-medium enterprises are still able to compete globally.

## Introduction

The Covid-19 pandemic and the government’s policies have resulted in a decrease in SMEs’ performance. This event is a big problem for SMEs due to the drastic decline in performance, and SMEs can no longer compete at the international marketing level. This research is fundamental to do if it will not cause bankruptcy from SMEs and will cause a more significant effect on the unemployed.

It is necessary to determine traditional financing for small- and medium-sized micro-enterprises already widely available. They can no longer support SMEs because of their complicated requirements, including funding derived from the combined capital (Rossi, 2015). Another problem faced by entrepreneurs is the difficulty of marketing SMEs’ products in the era of globalization not to afford to compete.

In the Covid-19 pandemic event, SMEs are generally only able to provide less than one-third of the required working capital, as additional money is needed (Waniak-Michalak et al., 2018). To innovative efforts to overcome the Covid-19 pandemic, this requires mediation from other parties to be more successful. In addition to financing issues, SMEs in Indonesia also have the advantage of competing with Malaysia and Thailand. According to Global Competitiveness Index 2015–2016 rankings and 2014–2015 comparisons, Indonesia ranked 37th while Malaysia is ranked 18th and Thailand at number 32 (Schwab, 2015).

It recognized that SMEs have a significant role in job creation, driving the economy’s wheels in the countryside, and providing a role in addressing unemployment and poverty. Nevertheless, a Covid-19 pandemic caused SMEs activity to become hampered and no longer able to run. Preliminary research (Sriyono et al., 2020) mentions several financing models for SMEs affected by Covid-19. Still, some of these models have not been implemented in the field correctly, so it is necessary to take an approach and assistance to SMEs and entrepreneurs to be able to conduct mutually beneficial cooperation.

This research aims to accelerate improving SMEs’ performance and competitiveness through non-banking financing so that SMEs can return to production. This research is very feasible to do. This research will find how to overcome SMEs’ problems about the lack of capital and difficulty in marketing experienced during the Covid-19 pandemic. Also, this research will make a significant contribution for SMEs to overcome the problems faced and for the government to process inputs that are used to provide policies on financing.

## Literature review

### SMEs based on green economy

Despite its many advantages, SMEs also face problems. The issue differs in each country, depending on how it affects performance (Noble, 2004). In advanced economies, small- and medium-sized enterprises are more related to intellectual property protection, such as patents on export products, while in developing countries such as Indonesia, the problems faced in the development of SMEs especially concerning aspects of business management capabilities and limited access to productive resources (Abraham & Schmukler, 2017).

The increase in knowledge and expertise is necessary to improve business management’s ability, especially in the era of globalization needed to encourage the competitiveness of international market-oriented SMEs’ products (Wiklund & Shepherd, 2005). There are many types of training provided by the government so far. Still, this activity is more routine, with material too theoretical, and a relatively short time to touch SMEs’ actual needs. The relationship between the market and its current performance with satisfactory performance will have a competitive advantage (Pelham, 2000).

These technological constraints can be caused by many factors, including capital limitations, to buy new machines to improve. Besides that improve the company’s performance, including production processes, limited information about the development of new technologies or production tools (Murphy, 1996), and hr limitations in operating new information technology machines or devices, making it challenging to make innovations products and production processes. In an era of free trade and global competition, modern technology’s use and mastery will become more important than natural resource factors to increase competitiveness and comparative advantage into a competitive advantage.

Also, SMEs whose activities are based on the green economy have long-term advantages, as conveyed in the study (Sriyono, 2015). Still, after that, they will gain customers’ trust because the products include a green economy.

### Financing from non-state budget

Other obstacles faced by SMEs are financial problems in the form of lack of capital and difficulty access (Angela, 2011) to obtain good capital in the form of credit (Arráiz et al., 2014) from financial institutions, especially banking (Beck et al., 2011). This problem is common in novice small-medium enterprises (SMEs), who do not have a business license, located in inland areas, with inadequate infrastructure conditions, making it difficult for financial institutions to reach actors with existing communication and transportation means.

It also is acknowledged that the difficulty of obtaining financial assistance from banks is due to several challenging requirements to meet by SMEs and the absence of a strong legal or regulatory basis for a high-risk business. SMEs’ financial structure is straightforward and more comfortable to meet (Beck et al., 2013) to obtain loans with easy terms, but it becomes difficult due to complicated regulations.

Financing originating from non-state budget can be various sources, can come through partnering between entrepreneurs and governments, or through partnerships between companies and SMEs or from funds from corporate social responsibility (CSR).

### Digital marketing

Competition pressures also arise due to the lack of accurate and up-to-date information about market opportunities in Bank Indonesia’s Role Small Medium Enterprise (SMEs). Profile in supporting the performance of micro, small, and medium enterprises development companies and abroad (Wiklund & Shepherd, 2005). Besides, in an era of openness and free trade, many countries in the world have agreed, such as agreements in the Asian Free Trade Agreement (AFTA), European Union (EU), and World Trade Organization (WTO), demand market openness in each country. Meanwhile, the rapid development of regulations issued by developed countries can hinder the growth of Indonesian SMEs from penetrating global markets, including a ban on the use of child labour, the necessity to pay attention to environmental preservation, and the protection of human rights.

## Research methodology

This research uses a qualitative approach (Creswell et al., 2007). Using interpretive methods (Lukka & Modell, 2010), this approach is very appropriate because this study interprets the results of an in-depth interview with key informants. This research also intended to get talks about something new little known and give the intricate details about the phenomenon of Covid-19 that is difficult to disclose by quantitative method (Strauss & Corbin, 2003).

The research focuses on understanding and analysing the informant’s opinion on SMEs’ problems due to Covid-19. The key informants in this study are several small-medium enterprises (SMEs). The use of crucial informants intended to allow researchers to obtain complete and more holistic information and some five experts in financing as a snowball for supporting information (Marshall, 1996).

The data collection process is through an in-depth interview (Moleong, 1996), documentation, and observation. This research data’s validity test is conducted with several stages: credibility and transferability (Senton, 2004). In-depth interviews are conducted with 25 SMEs owners established for 5 years with an interview time of 45–60 min on each informant. Besides, it also involved 5 SMEs’ financing experts who conducted 60-min and 75-min interviews. Interviews are conducted via teleconferences to all SMEs and experts. The question asked to SMEs is about the impact of Covid-19 on SMEs’ performance and the problems posed. Financial experts asked what model is suitable for SMEs during the Covid-19 pandemic and how to overcome current marketing problems. Credibility is using the source triangulation and triangulation method (Hussein, 2009). Transferability is by making detailed, systematic, and trustworthy research reports.

Qualitative data analysis is a continuous, repetitive, and constant effort consisting of three flows of activities that occur together (Muhson, 2006): reduction of research data, data display, and concluding.

## Result and discussion

### The impact of Covid-19 on SMEs

The emergence of the Covid-19 pandemic in the world has a significant impact in Indonesia, especially on SMEs’ entrepreneurs, resulting from these events, causing a decrease in SMEs’ performance and competitiveness. Therefore, in this study, interviews were conducted to what extent the impact of Covid-19 was. This research is a development from the initial research undertaken by (Sriyono et al., 2020). The first in-depth interview we did with SMEs around the company is shown below.

**Table:** 

In-depth interview and discussion	Response of small-medium enterprises (SMEs)	Triangulation test
How is the operation of SMEs during this Covid-19 pandemic?	*the business we do that automatically stops because no one buys, so we have no income. Finally, we are also unable to sell*	Then triangulation is done on SMEs located. Differently, the answer we get is the same that during pandemics, the SMEs cannot run their business
Whether you cannot try other companies that can be bought by the surrounding community, e.g., drinks or fried foods	*We have an idea, but it can't be executed because we don't have any more capital. The savings we have to survive to meet the blindness of family life.*	We are always thinking about wanting another business, we have a desire to sell simple food and drinks that are easy to sell, but we have no capital; our capital is up for daily life.

SMEs based on the green economy have a good performance and are responsible for environmental hygiene. Also, they have a significant role in reducing roasting and poverty and significantly contribute to national development. Nevertheless, the emergence of Covid-19 pandemic toughness owned by small-medium enterprises (SMEs) cannot withstand the decline in people’s purchasing power.

Unlike other large companies, unique SMEs’ characteristics require a particular model form that accommodates their uniqueness. Most SMEs are feasible but not bankable. Possible here, SMEs in business meet all the requirements to get additional capital from banking because SMEs can profit regularly from the business activities. But bankable, SMEs become not bankable because most SMEs do not meet the banking authorities’ administrative requirements, especially regarding the financial bookkeeping system. So, it needs a unique financing model that can accommodate the interests of SMEs.

For banks, the desire of SMEs to obtain financing becomes challenging to agree. This is because the banks are unable to monitor and focus on marketing, production, and managerial assistance. The policy and operational standards of procedures owned by banks do not allow to adopt all the wishes of SMEs, let alone coupled with the human power owned by banks which is very minimal when compared to SMEs that need to be served.

### Decreased performance and competitiveness of SMEs

**Table:** 

In-depth interview and discussion	Response of small-medium enterprises (SMEs)	Triangulation test
How do SMEs perform during the Covid-19 pandemic	*This Covid-19 is very troublesome for us because the food we do both in the store and in the market no one buys because they should not leave the house*.	Then triangulation is done on SMEs located. Differently, the answer we get is the same.

We also ask about the decrease in the competitiveness of SMEs due to the Covid-19 pandemic conditions.

In the era of industry 4.0, all entrepreneurs required to digitalization to compete, marketing or their part that has the role of selling all products owned, but as it is known that the resources owned by SMEs are less maximal so that they are not able to compete (Chadwick & Dabu, 2009). Therefore, breakthroughs are needed in digital marketing. Nowadays, the development of the science of marketing has entered the digital world so that digital marketing appears; digital marketing continues to develop through social media such as Instagram, Facebook, WhatsApp, Twitter, and many more.

To avoid a decrease in SMEs’ performance and competitiveness, it is now necessary to think that business innovation can be done through digital marketing (Coluccia et al., 2020).

### Competitiveness and digital marketing

**Table:** 

In-depth interview and discussion	Response of small-medium enterprises (SMEs)	Solution to that problem
How the marketing system has been done so far	During this time we sell our goods through the shop at home and directly to the traditional market. We’re still not able to do marketing online	The government should socialize with all SMEs to start learning to do digital marketing.
Is there a rescue programme for SMEs by providing capital derived from the particular budget for digital marketing	*? The Central Government policy is the first step in transferring the funds for Covid 19 but allocated first for health care. While the allocation assistance for additional capital has no specific policy, the government has not provided financing assistance directly because SMEs are challenging to apply for loans. After all, the Company is not bankable.*	Government policy must do to businesses because so far the procedure is only on macroeconomics.

Since the emergence of Covid-19 pandemics, for businesses or SMEs whose sales system conventional models will experience a decrease in purchasing power, shoppers cannot visit or go to shops, markets, and malls to buy goods because of lockdown policy. Therefore, innovation is needed so that SMEs can develop (Licuanan et al., 2015). This policy impacts marketing that still uses conventional models which will be difficult to market.

Therefore, it is necessary to find a breakthrough new model that is a digital marketing model; through this model, all products are offered through the Internet or social media. The problem is that many SMEs do not understand how to sell online, so education and mentoring are needed to make sales online.

### The government’s role on SMEs

The government as a regulator has an important role and obligation to help the problems caused to the impact of Covid-19 experienced by SMEs, because of whether the local government has ever gathered SMEs to provide loan information.

**Table:** 

In-depth interview and discussion	Response of SMEs	Solutions to that problem
How is the role of local governments in dealing with the impact of Covid-19	*The provincial government once provided information that it would be training assistance and self-help, but the implementation was not gradual, so we waited for the condition. We need more capital to create or open a new business that is currently trending, but we have not yet gained a way out*.	The government should provide socialization to SMEs to start educating business settlements due to Covid-19 continuously.

However, the government’s assistance pattern is directly in the field without continuous education and assistance. It can be understood because the government has enough resources to do so. Finally, this burden is all returned to SMEs and the community to assist in addressing the problem. Some educational institutions have also done assistance through entire lecture programmes and direct community service to SMEs. Still, this result is not maximal because of such activities integration between the trustees, educational institutions, and non-banking financial institutions.

### Financing for SMEs from companies through corporate social responsibility

In addition to the government, we also give to companies located around the location of small-medium enterprises (SMEs). It is known that every company has a corporate social responsibility programme.

**Table:** 

In-depth interview and discussion	Response of small-medium enterprises (SMEs)	Solution to that problem
Are the companies around here not assisting	The Company has assisted SMEs around the Company but has not been effective	The company should coordinate with the government and SMEs to make intensive cooperation
What cooperation do you do with the company	*we are involved in existing work; for example, we are given a project to choose suitable raw materials for the production process. We are given wages, those wages we use as additional capital, the additional capital we receive until now*	Companies can offer several types of cooperation with SMEs that are suitable and can be done by SMEs
Does this mean that you are receiving the current financing assistance in that way	*Yes, sir, at the end of the day, we get additional capital through that way. although the amount is not large, but we can get it regularly even if the funds are not large*	*The funds we get are uncertain depending on the type of work we can do, but the average is between IDR 150,000 – IDR 200,000 per week. So the total fund is around Rp 1,000,000*

Many alternatives can do to solve (Cusmano & Thompson, 2018) the SMEs’ problems. However, financing is a credit with a guarantee (Biernat-Jarka & Planutis, 2013) when granted on favourable terms. Based on the question, it can interpret that SMEs’ financing can be obtained from the fund even if the amount is not large; at least SMEs can do the activity again to do business, all of it depends on the company’s size. If the policy facilitates SMEs’ contact with the banks, then the financing problem will be more comfortable (Berger & Schaeck, 2011). Either through credit (Fatoki, 2014) for SMEs engaged in food stalls or small business drinks than with the fund can innovate other businesses that can be useful in Covid-19 pandemics, for example doing buy and sell masks, what makes drinks from spices that can add immune power to the body or household cuisine with system online and this will improve performance (Eniola & Ektebang, 2014).

### Expert opinion

**Table:** 

Discussion with financial expert	Financial expert opinion	Solution to that problem
What are their opinions about their problem	*The problem facing SMEs from the past until now is to remain in additional capital and marketing, especially directly coupled with the covid 19 pandemic outbreak, then the problem is getting bigger. In the past, access to financial institutions was also tricky because many SMEs businesses did not meet the Bank's requirements, namely the absence of orderly financial statements*	The conclusion, if you want to find additional capital that does not require requirements and low need can come from grants, people’s funds, or funds derived from corporate social responsibility of the company
How to implement the programme?	*It's not easy; just stay initiative is in the regulator only. if you want to improve SMEs welfare and solve covid 19 impacts, that's just not the case.*	This is finally back to the role of local leaders to initiate the programme. The government has the responsibility to solve these problems through several programmes to help the SMEs

Based on Sriyono et al.’s (2020) preliminary research, there are four financing models that SMEs can use for financing. However, of the four financing models, not all models can be implemented in Covid-19 pandemic conditions. Some models that exist in experts’ opinions are a model that does not provide onerous requirements for SMEs; for example, there must be collateral and bankable. Both of these conditions researchers believe will be suited to be met by SMEs, so severe orientation needs to be done (Kattenbach & Fietze, 2018) to implement.

The expert’s opinion concludes that the appropriate financing is financing derived from community social development fund, known as corporate social responsibility. Through the funding, it is expected that SMEs could accelerate innovation (Kuratko et al., 2011). The combination of the capital increase can also be done through venture capital, up to which venture capital is more the maximum result of institutional venture capital or venture capital firm venture (Rossi et al., 2020). Therefore, the approach that must be taken is encouraged by knowledge (Scuotto et al., 2017). However, venture capital’s role is very large in financing innovations that focus on new, small companies that innovate (Rossi & Martini, 2019). SMEs’ innovation can do if the SMEs’ owner has a transformational leadership to directly transfer ideas to employees (Moriano et al., 2014). If the financing is a combination of government and private parties, if supported by two parties, the programme’s sustainability will improve maximum (Prelipcean & Boscoianu, 2014). Business owners are directly responsible for the choice of financial sources. Different business-level managers have limited influence on this decision-making process. Therefore, the financial part has an essential effect in all decision-making scenarios.

## Conclusion

Based on the results of in-depth interview and search Library, Covid-19 pandemic has a big impact on SMEs namely the decrease in purchasing power, decreased revenue, and the decrease in competitiveness and SMEs do not have additional capital to do activities again. Therefore, it is necessary to provide the appropriate financing to overcome this, namely cooperation with the company to obtain funds derived from the fund of corporate social responsibility, because these funds do not require complex requirements. Also, severe education and assistance are needed for SMEs to change the sales system using digital marketing.

## Theoretical implication

Based on the results of in-depth interviews and searches Library, Covid-19 pandemic has a significant effect on SMEs: the decrease in purchasing power, decreased revenue, and the decline in competitiveness and SMEs do not have additional capital to do activities again. Therefore, it needs appropriate financing to overcome this, namely, cooperation with companies to obtain funds derived from corporate social responsibility funds, because the funds do not require complex requirements. Also, severe education and mentoring are needed for SMEs to change the sales system using digital marketing.

## Data Availability

Data is available on author and stored as private property.

## Abbreviations

SMEs: Small-medium enterprises
CSR: Corporate social responsibility
